# From Maternal Grazing to Barn Feeding During Pre-weaning Period: Altered Gastrointestinal Microbiota Contributes to Change the Development and Function of the Rumen and Intestine of Yak Calves

**DOI:** 10.3389/fmicb.2020.00485

**Published:** 2020-04-03

**Authors:** Zhanhong Cui, Shengru Wu, Shujie Liu, Lu Sun, Yuzhe Feng, Yangchun Cao, Shatuo Chai, Guomo Zhang, Junhu Yao

**Affiliations:** ^1^College of Animal Science and Technology, Northwest A&F University, Yangling, China; ^2^Qinghai Academy of Animal Husbandry and Veterinary Sciences, Qinghai University, Xining, China; ^3^Datong Yak Breeding Farm of Qinghai Province, Xining, China

**Keywords:** yak calves, feeding strategies, gastrointestinal microbiota, mRNA sequencing, 16S rRNA gene sequencing, growth performance

## Abstract

Understanding the altered gastrointestinal microbiota is important to illuminate effects of maternal grazing (MG: maternally nursed and grazed) and barn feeding (BF: supplied milk replacer, starter feed, and alfalfa hay) on the performance and immune function of yak calves. Compared with the MG group, the significantly increased body weight, body height, body length, chest girth, and organ development of liver, spleen, and thymus were identified in the BF group, which were resulted from the significantly increased dry matter intake, increased concentrations of propionate, butyrate, isobutyrate, and valerate, increased ruminal pectinase, duodenal α-amylase, jejunal α-amylase and trypsin, and ileal trypsin, and promoted gastrointestinal epithelial development. Furthermore, genera of *Sharpea*, *Sphingomonas*, *Atopobium*, *Syntrophococcus*, *Clostridium*_*XIVb*, *Acinetobacter*, *Oscillibacter*, *Dialister*, *Desulfovibrio*, *Bacteroides*, *Lachnospiracea*_*incertae*_*sedis*, and *Clostridium*_*sensu*_*stricto*, which were involved in utilization of non-fibrous carbohydrate and further beneficial to improve the gastrointestinal digestion, development, and immune functions, were significantly increased in the BF group. Meanwhile, the significantly enhanced ruminal epithelial immune functions and intestinal immune functions based on enhanced ruminal immune related pathway, duodenal IL-1β, jejunal IL-1β, IL-2, TNF-α, and IFN-γ, and ileal IL-1β were identified in the BF group, which also may induced by the increased abundance of gastrointestinal microbiota. Overall, barn feeding significantly increased the diversity of species and abundance of microbes which used different carbohydrates and further benefit to the growth and immune function of yak calves.

## Introduction

The yak, which is the major indigenous ruminant on the Qinghai-Tibetan Plateau in China, has been grazed and used by local herdsmen for milk, meat, and fuel from feces for several centuries (Xue et al., 2017). Calves are important for the sustainable development of the yak industry in the Qinghai-Tibetan Plateau, so the quality of calf rearing directly determines the performance of adults (Long et al., 2008). The early life is a critical period for the developmental plasticity of young ruminants and can have a long-term impact on various biological functions (Baldwin et al., 2004; Soberon and Van Amburgh, 2013). The pre-weaning period for yak calves mainly occurs during maternal grazing and nursing, which were not beneficial to the oestrs and mating of female yaks or the survival and growth of calves (Long et al., 2008; Damián et al., 2017). New ways of feeding during the pre-weaning period (early life) need to be explored for yak calves, among which barn feeding with mixed rations of available roughage, grains, and milk replacer is an alternative to natural maternal grazing and nursing.

Adequate nutrition during early life is beneficial to gastrointestinal development, immunity, and the subsequent functional transition from metabolizing the lactose from milk to the volatile fatty acids (VFAs) from a solid diet (Liu et al., 2017; Saro et al., 2018; Yang et al., 2018); this transition could result in tremendous gastrointestinal metabolism and immunity alterations for the growth rate of calves (Shade et al., 2012). Many previous studies have proved that the growth performance and carcass characteristics of yaks could be improved under barn feeding when compared with maternal grazing conditions (Dong et al., 2006; Raghuvansi et al., 2007; Baruah et al., 2012; Chen et al., 2015). Meanwhile, some studies have also proved that the maternal milk intake could be beneficial to the growth and the development of organ and immune function (Schack-Nielsen and Michaelsen, 2007). So far, few studies have simultaneously compared the growth, development, and immune function of yak calves under maternal grazing with milk and fresh grass versus barn feeding with mixed rations of available roughage, grains, and milk replacer, which could add our knowledge of yak calves breeding during the pre-weaning period.

The gastrointestinal microbiota can be manipulated by altered feeding strategy and subsequently altered nutritional supplementation to improve productivity and health (Ben Salem et al., 2005; Abecia et al., 2014). Specifically, the colonization of the gastrointestinal microbiota of newborn ruminants starts during birth and continues through successive waves of colonization and community changes until the microbiota reaches a stable state later in life, indicating that the gastrointestinal microbiota during early life is easily affected by altered diets and environmental exposures (Rey et al., 2014; Yáñez-Ruiz et al., 2015). Moreover, Receipt of breast milk was the most significant factor associated with the microbiome structure, and the cessation of breast milk resulted in faster maturation of the gut microbiome, which could further influence the subsequent immune and growth performance (Stewart et al., 2018). Gastrointestinal microbiota is integral to feed digestion, nutrient absorption and metabolism, immune response, and gastrointestinal development in ruminants (Morgavi et al., 2015). Hence, barn feeding with available roughage, grains, and milk replacer during early life has the potential to influence the gut microbiota and further results in tremendous gastrointestinal functional alterations. In the present study, the effect of two strategies during early life on the gastrointestinal microbiota of yak calves was also evaluated, with the aim of further illuminating the roles of altered gastrointestinal microbiota in regulating the gastrointestinal function and development of yak calves, which were further beneficial to their growth and development.

## Materials and Methods

### Animals and Experimental Design

All the yak calves and experimental protocols in this study were approved by the Institution Animal Care and Use Committee of the Northwest A&F University (protocol number NWAFAC1118).

Before the commencement of the trial, all yak calves were fed with the milk by maternal nursing only in Datong Yak Breeding Farm of Qinghai Province. In brief, the experiment was performed in Datong Yak Breeding Farm, where the average temperature of daytime were 18°C from the barn and 10°C from the pasture. Meanwhile, the dry matter/m^2^, pasture height, and leaf/stem of pasture where the grazing group yaks were kept were also measured and presented as following: dry matter/m^2^: 89 g/m^2^, pasture height: 10.5 cm, and leaf/stem (Dry matter basis): 51.73 g/m^2^ vs. 31.27 g/m^2^. A total of ten 30-day-old male yak calves (34.86 ± 2.06 kg, no significant differences between two groups) with similar body condition were randomly assigned to two groups with five calves per group. The maternal grazing (MG) group was maternally nursed and grazed, and the barn feeding group was supplied milk replacer, starter feed, and alfalfa hay. The yak calves in the maternal nursing group had access to fresh grass and yak milk.

Briefly, The MG yak calves were allowed to graze a rangeland for a period of 8 h. Water was offered *ad libitum* twice a day at 0800 and 1600 h. Specifically, the experiment was performed from July to October and lasted for 90 days, allowing for the sufficient grazing of fresh grass. Moreover, at the last day of each period (10-day intervals as a period), the fresh grass and the yak milk were collected and provided to the yak calves, and a total of nine measures of the feed intakes for each of the calves were recorded and used to calculate the dry matter intake. The yak calves in the barn feeding group were housed in a barn and kept in individual pens (7 m × 4 m). The pens included a sawdust-bedded pack area and a feed lane equipped with an automatic cable scraping system. In addition to free access to starter feed and alfalfa hay, all yak calves in barn feeding group were supplied with milk replacer reconstituted from 100 to 350 g milk replacer powder (the supplementation of milk replacer were increased along with the increasing body weight) dissolved in 1 L 60°C water twice per day at 08:00 and 16:30. Water was supplied *ad libitum* to the yak calves during the experimental period. During the experimental period, the feed offered was recorded at the last 3 days of each period (10-day intervals as a period), and the residue was also collected at the last 3 days of each 10 days, then pooled and weighed at 3-day intervals for the calculation of the averaged daily feed intake over the 3 days. This approach resulted in a total of nine measures of the feed intakes for each of the calves over the whole period, and the mean of those nine intakes was used an individual replicate for the statistical analysis on the difference of feed intake between two treatments.

Feed (including the alfalfa and starter feed) offered was adjusted daily to ensure at least 10% orts. Feed offered and refused by each calves were weighed and recorded at the last day of feeding experiment. Meanwhile, the daily intake was calculated for further analysis of DMI (overall considering the dry matter intake of milk replacer, alfalfa, and starter feed). After the feeding experiment, the yak calves were weighed, and their body size indexes, including the body height, body length and chest girth, were recorded.

The details of the nutrient composition of the fresh grass, starter feed, alfalfa hay, and milk replacer were measured in accordance with the previous described methods (Xu et al., 2018) and were given in Supplementary Tables S1, S2. Briefly, All substrates were dried in a forced-oven at 65°C for 48 h, ground through a 1-mm screen Wiley mill (Tecator 1093; Tecator AB, Höganäs, Sweden), and then stored at 4°C until further analysis. According to AOAC procedure (AOAC, 2005), the samples were analyzed for dry matter (DM, 105°C), crude protein (CP, # 988.05), ether extract (EE, # 922.06), ash (# 942.05), and acid detergent fiber (ADF, # 973.18). The neutral detergent fiber (NDF) was analyzed using a method by Van Soest et al. (1991). Meanwhile, the nutrient composition of yak milk was also measured in accordance with the previous description (da Silva et al., 2016) and given in Supplementary Table S2.

### Sample Collection and Determination of Organ Index

After the 90-day feeding period, blood samples of all yak calves were collected before morning feeding, and the plasma samples were prepared. Then these calves were weighed, euthanized by exsanguination after intravenous administration of 10% chloral hydrate solution (100 mg chloral hydrate/kg body weight; Sigma, United States) and immediately dissected. The liver, thymus, spleen, and pancreas were collected and weighed immediately. Organ indexes were expressed relative to body weight (kg of organ/kg of body weight). Then, 30 cm^2^ epithelial tissue samples of the dorsal rumen at the same position were also collected for direct measurement of the papillae length and width of rumen epithelium and the thickness of rumen base (Ishii et al., 2005). Furthermore, following removal of the intestinal contents to prevent contamination, the middle complete duodenal, jejunal, and ileal segments were collected in lengths of 3 cm and fixed in 10% buffered formalin, where they remained for at least 48 h for further histological processing. Briefly, to ensure that the sampling sites were consistent, we segmented the intestine into duodenum, jejunum, and ileum using the following criteria: the duodenum tissue was the first 15 cm of the small intestine beginning at the pyloric sphincter; the ileum tissue was the distal 15 cm portion of the small intestine that ended at the ileocecocolic junction. About 15 cm from the middle of the small intestine was sampled as the jejunum tissue. Then the middle complete intestinal segments in lengths of 3 cm were separately collected for intestinal morphology. And the residual 12 cm sampled duodenal, jejunal, and ileal segments were used to collect duodenal, jejunal, and ileal mucosal samples. The duodenal, jejunal, and ileal mucosal samples at the same position were used to measure the activities of gastrointestinal immune cytokines, and the rumen dorsal epithelial tissue samples at the same position were used for further qRT-PCR and RNA sequencing. All tissue samples were also taken from the same location in each animal. Briefly, the duodenal, jejunal, and ileal sections were cut along the dorsal line, and the contents were emptied. The fundic region of these tissues was washed with ice-cold 0.9% saline, and the mucosa was scraped using a sterile glass slide. Specifically, in order to avoid the potential influence of Payer’s patch on the subsequent measurement, the presence of Payer’s patch was firstly confirmed and only the patch-free segment of jejunum were used for sample collection and further analysis. Meanwhile, rumen fluid was collected and strained through four layers of sterile cheesecloth. The pH of the rumen fluid was measured immediately with a mobile pH meter (HI 9024C; HANNA Instruments, Woonsocket, RI, United States); meanwhile, another 5 mL of rumen fluid was collected for VFA and NH3-N analyses. Specifically, a solute with metaphosphoric acid and crotonic acid was added to 2 mL of these 5 mL rumen fluid samples for further analyses of the VFA concentrations. Moreover, rumen fluid and the abomasal, duodenal, jejunal, and ileal content samples were also collected for further 16S rRNA gene sequencing and digestive enzymes analyses. The duodenal, jejunal, and ileal segments and the rumen dorsal epithelial tissue samples, which were fixed in 10% buffered formalin, were stored at 4°C until the analysis of rumen and intestinal morphology, and the other samples were firstly stored in liquid nitrogen for 24 h and then stored in −80°C until analyses.

### Determination of Rumen and Intestinal Morphology

Epithelial tissue (30 cm^2^) of the dorsal rumen, as well as the middle complete duodenal, jejunal, and ileal segments, which were fixed in 10% buffered formalin, were used for the analysis of rumen and intestinal morphology. After being fixed, the samples were gradually dehydrated by using different concentrations (60, 70, 80, 90, and 100%) of ethanol. Then, the rumen and intestinal samples were cut and inserted into cassettes, which were embedded in liquid paraffin. Next, 5 μm paraffin sections were cut using the microtome and stained with hematoxylin and eosin. The papillae length and width of rumen epithelium, the thickness of the rumen base, and the height and crypt depth of the intestinal villus were determined using a phase contrast microscope (Nikon, Japan) (Wu et al., 2018).

### Determination of VFA in Rumen Fluid

For VFA and NH_3_-N measurements, the rumen fluid was centrifuged at 13,000 × *g* for 10 min. VFAs were analyzed by an Agilent 6850 gas chromatograph (Agilent Technologies Inc., Santa Clara, CA, United States) equipped with a polar capillary column (HP-FFAP, 30 m × 0.25 mm × 0.25 μm) and a flame ionization detector, as previously described (Xue et al., 2017). NH_3_-N in the supernatant was quantified using a continuous flow analyzer (SKALAR San++, Skalar Co., Netherlands).

### Measurement of the Activities of Gastrointestinal Digestive Enzymes and Immune Cytokines

The activities of gastrointestinal digestive enzymes, including carboxymethyl cellulase, xylanase, and pectinase in the rumen, pepsin, and chymosin in the abomasum, and α-amylase, trypsin, and lipase in the duodenum, jejunum, and ileum, were measured by spectrophotometric methods according to the manufacturer’s instructions (Jiancheng Biological Engineering Research Institute, Nanjing, China). Moreover, the secreted immunoglobulin A (sIgA), interleukin-1β (IL-1β), interleukin-2 (IL-2), interleukin-4 (IL-4), interleukin-10 (IL-10), tumor necrosis factor-α (TNF-α), and interferon-γ (IFN-γ) content of mucous membrane samples from the duodenum, jejunum, and ileum were measured by enzyme-linked immunosorbent assay (ELISA) kits for cattle (YuanMu Biological Technology Co., Ltd., Shanghai, China).

### Microbial DNA Extraction, 16S rRNA Gene Amplification of the V3 + V4 Region, Sequencing, and Bioinformatics Analysis

The 8 rumen fluid samples were used for DNA extraction using the CTAB-based method in accordance with Henderson et al., 2013. Meanwhile, the eight jejunal content and eight ileal content samples of yak calves from two different treatments were used for DNA extraction using a QIAamp DNA Stool Mini Kit (Qiagen, Germany). The integrity of the DNA was assessed using 1% agarose gel electrophoresis and the purity was assessed from the 260:280 nm ratio (>1.8) using a NanoDrop ND2000 spectrophotometer (Thermo Scientific, United States), and the DNA was stored at −80°C until it was used in sequencing analysis. Only the DNA samples with an optical density ratio at 260/280 nm > 1.8 (Supplementary Table S3) and with ideal integrity (assessed by gel electrophoresis) were used in further analyses.

The amplicon library was prepared by polymerase chain reaction amplification of the V3–V4 region of the 16S rRNA gene using the primer set 341F (5′-CCTAYGGGRBGCASCAG-3′) and 806R (5′-GGACTACNNGGGTATCTAAT-3′) with barcodes (Yu et al., 2005; Sundberg et al., 2013). PCR reactions were performed in triplicate 20 μL mixture containing 4 μL of 5 × FastPfu Buffer, 2 μL of 2.5 mM dNTPs, 0.8 μL of each primer (5 μM), 0.4 μL of FastPfu Polymerase, and 10 ng of template DNA. Amplicons were extracted and purified from 2% agarose gels using the AxyPrep DNA Gel Extraction Kit (Axygen Biosciences, United States) and were quantified using QuantiFluor^TM^ -ST (Promega, United States).

The 16S rRNA gene amplicons were used to determine the diversity of and to perform structural comparisons of the bacterial species present in each of the samples using paired-end sequenced (2 × 250) on an Illumina HiSeq 2500 platform according to the standard protocols. The sequence data were deposited and are available in the Sequence Read Archive (SRA) of NCBI with the accession project numbers PRJNA543073.

Raw fastq files were demultiplexed using the barcode sequence with the exact barcode matching parameter. Quality-filtering of raw tags were performed using Trimmomatic (version 0.36) (Bolger et al., 2014) with the following criteria: (i) bases off the start and end of a read below a threshold quality (Score < 2) were removed (ii) The reads were truncated at any site receiving an average quality score < 2 over a 4 bp sliding window, discarding the truncated reads that were shorter than 100 bp.

Paired-reads were merged using USEARCH (version 9.2.64)^1^ (Edgar and Flyvbjerg, 2015) with the default parameters. The primer sequences were identified and removed from the merged reads by using the subcommand “search_pcr” of USEARCH. Then reads which could not be merged were discarded and the merged reads with more than two nucleotide mismatch in primer matching were removed. Then the singletons reads were eliminated for the following analysis. These sequences were classified into operational taxonomic units (OTUs) at an identity threshold of 97% similarity using UPARSE software (Edgar, 2013). For each OTU, a representative sequence was screened and used to assign taxonomic composition by comparison with the RDP 16S Training set (v16) and the core set using the SINTA (Usearch V9.2.64) and PyNAST programed algorithms (Caporaso et al., 2010; Edgar, 2016) to identified the phylum, class, order, family, and genus. Subsequent analysis of alpha and beta diversity was performed based on the output of this normalized data by separately using USEARCH alpha_div (Edgar, 2010) and UniFrac metrics (Lozupone and Knight, 2005) in QIIME (version 1.9.1). The taxon abundance for each sample was determined according to phylum, class, order, family, and genus. The microbiota were compared for beta diversity using the distance matrices generated from weighted UniFrac analysis and principal coordinated analysis (PCoA) and ANOMIS analysis. Linear discriminant analysis (LDA) effect size (LEfSe) analysis and Mann-Whitney *U* test was performed to estimate the effect size of species that contributed to the differences between the samples. The *P* value was set as <0.05, and the threshold of the LDA score was set at a default value of 2.0.

### RNA Isolation From the Rumen Epithelium, Sequencing, and Bioinformatics Analysis

Total RNA from the rumen epithelial tissue samples of 10 yaks was extracted using TRIzol reagent (Invitrogen, CA, United States). Specifically, DNase I was used during the RNA isolation process to avoid contamination with genomic DNA. The quantity and purity of the total RNA was analyzed by a NanoDrop^®^ ND-1000 spectrophotometer (Thermo Scientific, MA, United States), and the integrity of the RNA was assessed with the Bioanalyzer 2100 and RNA Nano6000 LabChip Kit (Agilent, CA, United States). Only samples that had an OD260/280 > 1.8, OD260/230 > 2.0, and an RNA Integrity Number > 7.0 were used for further sequencing.

Approximately 3 μg of total RNA from each sample was used to prepare an mRNA library according to the Illumina^®^ TruSeq^TM^ RNA sample preparation protocol. Then, the paired-end sequencing (2 × 125 bp) was performed on an Illumina HiSeq 2500 at the Novogene Bioinformatics Institute (Beijing, China). The 125 bp paired-end raw reads were firstly processed through SOAPnuke filte to obtain the clean data, by removing the reads that contain sequencing adapter contaminations or poly-N and the low quality reads whose *Q* value were less than 20. At the same time, Q20 and Q30 of the clean data were calculated (Supplementary Table S4) and all of them were in good quality (with Q20 > 97% and Q30 > 92%). The index of the reference genome was built using Bowtie v2.2.3 (Langmead and Salzberg, 2012), and the sequences were aligned to the yak genome (Bos mutus, assembly BosGru_v2.0) using HISAT (Kim et al., 2015). Sequence segments were spliced and annotated, and transcript expressions were calculated by RSEM (Li and Dewey, 2011). Fragments per kilobase of exon per million mapped reads (FPKM) was employed to quantify the gene expression. Based on negative binomial distribution, differentially expressed genes (DEGs) were screened out by using DESeq with an adjusted *P* < *0.05* and fold change >2 or <0.5. Kyoto Encyclopedia of Genes and Genomes (KEGG) pathway enrichment analysis for the DEGs was performed by using KOBAS software (Xie et al., 2011). *P* < 0.05 was used to define KEGG pathways as significantly enriched.

### qRT-PCR Analysis

Approximately 1 μg of total RNA was reverse transcribed using a PrimeScript^TM^ RT reagent kit with gDNA eraser (TaKaRa, Dalian, China). qRT-PCR was performed using a SYBR^®^ Green PCR Master Mix (TaKaRa, Dalian, China). A 20 μL PCR mixture was quickly prepared. Primers for *GAPDH* (internal control gene) and five tested mRNAs (DEGs selected by RNA sequencing) were designed using Primer-BLAST^2^ and are listed in Supplementary Table S5 The PCR was conducted in an iCycler iQ5 multicolor real-time PCR detection system (Bio-Rad Laboratories) and programed as follows: 95°C for 10 min; 40 cycles of 95°C for 10 s, 60°C for 30 s, and 72°C for 30 s; and 72°C for 5 min. All samples were examined in triplicate. All data were analyzed using the 2^–ΔΔ*Ct*^ method (Livak and Schmittgen, 2001).

### Statistical Analysis

In the present study, all data were tested and were all presented as normal distribution. The differences of DMI between yaks from two groups were further analyzed using the MIXED procedure of SAS (SAS Institute Inc., Cary, NC, United States, 2007) using the following model: Yij = μ + Di + Tj + TD + εij, which considering the significant differences of these indices induced by the time effect in the same treatment. In brief, Yij is the response variable, μ is the overall mean, Di is the fixed effect of treatment (*i* = maternal grazing or barn feeding), Tj is the fixed effect of time (10 days as a unit) of the experiment, TD is the interaction of dietary and time, and εij is the residual error. If a significant diet and time effect was observed, the significance between the treatment and time differences was separately identified by Tukey’s test multiple comparisons test. All data are expressed as the means with the standard error. Differences were considered to be statistically significant at *P* < 0.05.

At the beginning of the experiment, the initial body weight, body height, body length, and chest girth were tested by students’ *t* test, and no significant differences were identified and the initial indices were almost the same. Hence, for other indices except for the DMI data, analyses were performed using Student’s *t* test with SPSS 21.0 software with replicates as experiment units, and differences were considered statistically significant at *P* < 0.05.

## Results

### Barn Feeding of Yak Calves During Early Life Significantly Promoted Growth and Organ Development

The significant differences between the treatments in the daily DMI of yak calves were identified in the present study, where the increased intake was found for calves on barn feeding group (Table 1). Meanwhile, the DMI of the calves were gradually increased with the gain of body weight of the calves, and the differences between the treatments in feed intake were persistent over the whole period (Supplementary Figure S1). Herein, compared with the yaks in the maternal grazing group, the yaks in the barn feeding group showed significantly increased body weight, body height, body length, and chest girth (Table 1). Additionally, the weight of the liver, spleen, and thymus and the organ index of the thymus and pancreas were also significantly increased in the barn feeding group, while the weight of the pancreas and organ index of the liver and spleen were not altered (Table 1).

**TABLE 1:** Effects of two feeding strategies (maternal grazing and barn feeding) during early life on the dry matter intake, body weight and body size traits, organ weight, and organ index of yak calves.

Items		Treatments		*P*-value
		Barn feeding	Maternal grazing	
Growth performance	Body weight (kg)	64.5 ± 6.67	87.9 ± 1.60	0.005
	Chest girth (cm)	107.3 ± 4.65	116.2 ± 2.90	0.021
	Body length (cm)	86.0 ± 3.46	109.2 ± 3.40	< 0.001
	Body height (cm)	77.3 ± 6.95	95.6 ± 2.30	0.010
	Dry matter intake (g)	1396 ± 463	1201 ± 380	< 0.001^1^
Organ weight	Liver weight (g)	1391 ± 32.7	1058 ± 76.6	0.001
	Spleen weight (g)	238 ± 34.2	151 ± 14.0	0.003
	Thymus weight (g)	253 ± 11.4	105 ± 10.5	< 0.001
	Pancreas weight (g)	50.6 ± 6.79	50.8 ± 2.71	0.939
Organ index	Liver index (× 10^–2^)	1.58 ± 0.025	1.61 ± 0.106	0.722
	Spleen index (× 10^–2^)	0.27 ± 0.037	0.228 ± 0.005	0.050
	Thymus index (× 10^–2^)	0.29 ± 0.016	0.16 ± 0.008	< 0.001
	Pancreas index (× 10^–2^)	0.057 ± 0.008	0.077 ± 0.001	0.002

*^1^ the *P* value < 0.001 for dry matter intake analysis indicated that the *P*_*diet*_ < 0.001, *P*_*time*_ < 0.001, *P*_*diet* × *time*_ < 0.001.*

### Significantly Enhanced Rumen Fermentation, Intestinal Digestion, and Gastrointestinal Development Were Identified in the Yak Calves in the Barn Feeding Group

The rumen fermentation characteristics of yak calves under the grazing and barn feeding conditions are presented in Table 2. The pH and NH_3_-N showed no differences between the different feeding groups. The total VFA concentration was significantly higher in the barn feeding group than in the grazing group. Significantly higher concentrations of propionate, butyrate, isobutyrate, and valerate were also identified in the barn feeding group, whereas acetate and isovalerate were not significantly altered in the present study. Furthermore, the ratio of acetate/propionate and acetate/total VFA were significantly decreased in the barn feeding group, while the ratio of butyrate/total VFA, isobutyrate/total VFA, and valerate/total VFA were all significantly increased in the barn feeding group. Meanwhile, the activities of ruminal and abomasum enzymes in the yaks in the two groups are presented in Table 2. Ruminal pectinase was significantly increased in the barn feeding group (Table 2). Moreover, the ruminal morphology, including the papillae length and width of rumen epithelium, were significantly increased in the barn feeding group, while the thickness of the rumen base was not affected (Table 3).

**TABLE 2:** Effects of two feeding strategies (maternal grazing and barn feeding) during early life on the ruminal fermentation characteristics, as well as the ruminal, abomasum, and intestinal enzyme activities of yak calves.

Items		Treatments		*P*-value
		Barn feeding	Maternal grazing	
Ruminal fermentation characteristics	Total VFA content	57.8 ± 3.60	67.1 ± 1.82	0.002
	Acetate/Propionate	4.17 ± 0.10	3.65 ± 0.27	0.011
	Acetate/Total VFA	0.71 ± 0.012	0.63 ± 0.016	< 0.001
	Propionate/Total VFA	0.17 ± 0.001	0.17 ± 0.009	0.580
	Butyrate/Total VFA	0.07 ± 0.009	0.13 ± 0.006	< 0.001
	Isobutyrate/Total VFA	0.01 ± 0.002	0.02 ± 0.002	0.035
	Valerate/Total VFA	0.014 ± 0.101	0.024 ± 0.004	< 0.001
	Isovalerate/Total VFA	0.026 ± 0.004	0.026 ± 0.002	0.482
	Ammonia nitrogen, NH_3_N (mg/dL)	6.92 ± 0.63	6.56 ± 0.83	0.485
	pH	6.84 ± 0.47	7.17 ± 0.10	0.733
Ruminal enzymes	Carboxymethyl cellulase (U)	82.9 ± 4.94	79.3 ± 3.14	0.229
	Pectinase (U)	245 ± 35.3	174 ± 8.8	0.006
	Xylanase (U)	13.8 ± 1.43	12.6 ± 1.42	0.321
Abomasum enzymes	Pepsin (U)	34.4 ± 3.14	30.3 ± 1.69	0.045
	Chymosin (U)	84.5 ± 2.92	81.8 ± 7.28	0.475
Duodenum enzymes	Alpha amylase (U)	45.5 ± 3.33	40.3 ± 1.41	0.022
	Trypsin (U)	9.59 ± 0.57	13.57 ± 1.29	0.005
	Lipase (U)	4.56 ± 0.66	6.41 ± 0.95	0.020
Jejunum enzymes	Alpha amylase (U)	42.2 ± 2.56	35.7 ± 2.00	0.004
	Trypsin (U)	13.04 ± 1.16	8.81 ± 2.15	0.021
	Lipase (U)	6.45 ± 1.42	10.03 ± 1.19	0.005
Ileum enzymes	Alpha amylase (U)	34.9 ± 3.01	40.8 ± 1.60	0.010
	Trypsin (U)	4.34 ± 0.74	3.76 ± 0.64	0.259
	Lipase (U)	5.38 ± 0.69	7.14 ± 1.98	0.103

**TABLE 3:** Effect of two feeding strategies (maternal grazing and barn feeding) during early life on the ruminal and intestinal morphology of yak calves.

Items		Treatments		*P*-value
		Barn feeding	Maternal grazing	
Rumen	Papillae width (μm)	1034 ± 29.9	826 ± 28.9	< 0.001
	Papillae length (μm)	529 ± 12.1	429 ± 10.1	< 0.001
	Muscle thickness (μm)	2080 ± 27.9	2023 ± 99.4	0.337
Duodenum	Villus length (μm)	1044 ± 44.8	854 ± 25.2	< 0.001
	Crypt depth (μm)	630 ± 12.9	427 ± 8.9	< 0.001
Jejunum	Villus length (μm)	1197 ± 19.9	951 ± 20.8	< 0.001
	Crypt depth (μm)	636 ± 27.9	478 ± 18.1	< 0.001
Ileum	Villus length (μm)	1147 ± 32.7	971 ± 34.7	< 0.001
	Crypt depth (μm)	531 ± 16.8	341 ± 17.7	< 0.001

**FIGURE 1:**
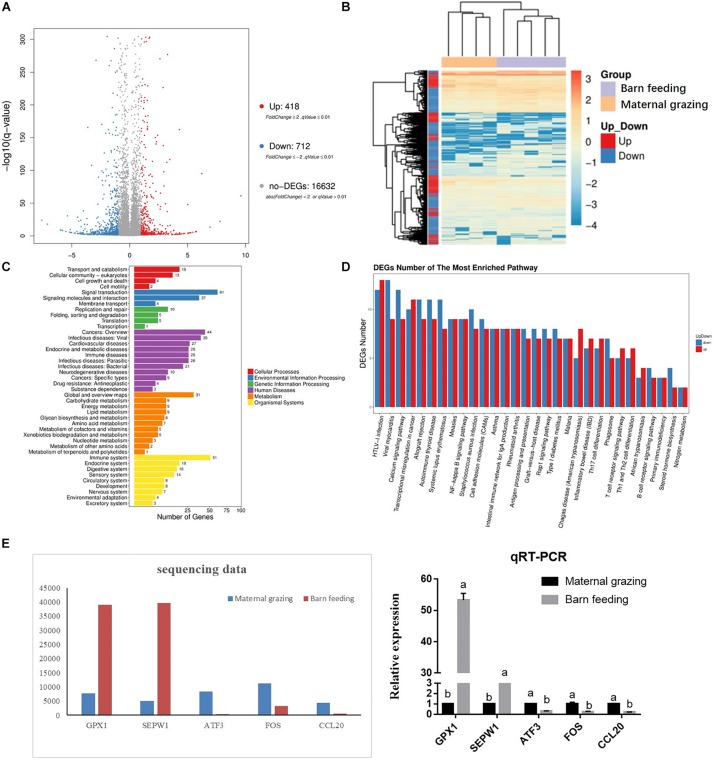
Differentially expressed mRNAs in the rumen epithelium of yak calves from two different feeding strategies (maternal grazing and barn feeding) groups. **(A)** The volcano plot shows the significantly differentially expressed genes (barn feeding group vs maternal grazing group). **(B)** The heatmap of significantly differentially expressed genes. **(C)** Pathway classification based on the differentially expressed genes between the barn feeding group and maternal grazing group. **(D)** Significantly enriched pathways based on differentially expressed genes between the barn feeding group and maternal grazing group. **(E)** Sequencing of five selected DEGs verified by qRT-PCR.

RNA sequencing was performed to study the effect of two different feeding strategies on the mRNA profiles of the rumen epithelium. As a result, 418 upregulated and 712 downregulated genes were identified in the yaks of the barn feeding group compared with the yaks of the grazing group (Figure 1A). The gene expression profiles of these 1130 DEGs were significantly different between the grazing and barn feeding groups (Figure 1B). Based on functional gene clustering, pathways involved in carbohydrate metabolism, energy metabolism, lipid metabolism, glycan biosynthesis and metabolism, and amino acid metabolism were affected by the two different feeding strategies (Figure 1C). Meanwhile, we further listed the differentially expressed genes involved in these metabolism process in Supplementary Table S6. Specifically, we can find that the ACSS, CA4, CA8, and NDUFA4L2, which are involved in the carbohydrate metabolism (acetyl-CoA biosynthetic process) and energy metabolism (NADH metabolism), were all significantly increased in the barn feeding group, which indicated that the metabolism of these nutrients and their absorption were all enhanced in the barn feeding groups.

The intestinal enzymic activities in two groups were also measured. Compared with the yaks in the grazing group, the yaks in the barn feeding group had significantly increased α-amylase in the duodenum, α-amylase and trypsin in the jejunum, and trypsin in the ileum (Table 2). In contrast, the duodenal trypsin and lipase, jejunal lipase, and ileal lipase were significantly decreased in the barn feeding groups (Table 2). Furthermore, the intestinal morphology was also measured. In the barn feeding group, the villus height and crypt depth of the duodenum, jejunum, and ileum in the barn feeding group were also significantly increased in the present study (Table 3).

### Barn Feeding Was Beneficial to the Gastrointestinal Immune Function of Yak Calves

The gastrointestinal immune functions were also evaluated. In the rumen, the results of the KEGG analyses based on the identified DEGs indicated that most of the altered genes were involved in the regulation of immune functions, which could be concluded from abundant genes enriched in biological processes involved in disease occurrence (Figure 1C). The functional clustering of organismal systems also indicated that the immune system was the most affected system (Figure 1C). Furthermore, the KEGG enrichment analyses further identified 30 significantly enriched KEGG pathways, and most of these significantly enriched pathways were related to the immune regulation process (Figure 1D). Meanwhile, we also listed the differentially expressed genes involved in these immune related pathways in Supplementary Table S7. In brief, we can find that the barn feeding could meanwhile significantly increased the genes expression involved in the anti-inflammation and pro-inflammatory regulation related pathways and the immune cell development and differentiation related pathways. Moreover, five of these DEGs were further verified by qRT-PCR, and the results indicated that the expression of the selected mRNAs detected by qRT-PCR was consistent with the mRNA sequencing data (Figure 1E).

**FIGURE 2:**
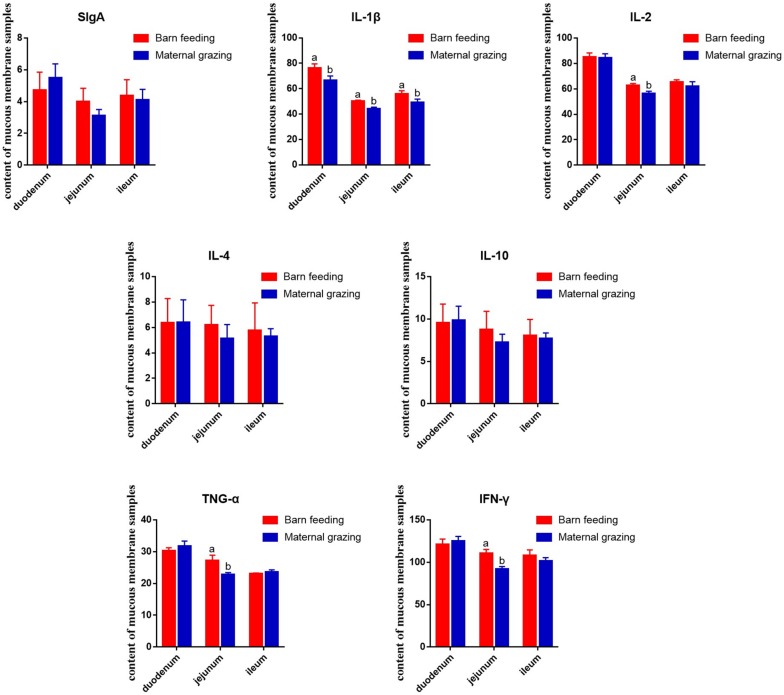
Effect of two different feeding strategies (maternal grazing and barn feeding) during early life on the sIgA and immune cell cytokine content of mucous membrane samples from the duodenum, jejunum, and ileum of yak calves.

Furthermore, based on the identified altered immune function in rumen, the intestinal immune functions were also identified. As results, the duodenal IL-1β, jejunal IL-1β, IL-2, TNF-α, and IFN-γ, and ileal IL-1β were significantly increased in the barn feeding group compared with the grazing group (Figure 2). However, sIgA and other immune cell cytokines in different intestinal segments were not significantly altered (Figure 2).

### Diversity and Abundance Changes of Gastrointestinal Microbiota Response to Two Different Feeding Strategies

**FIGURE 3:**
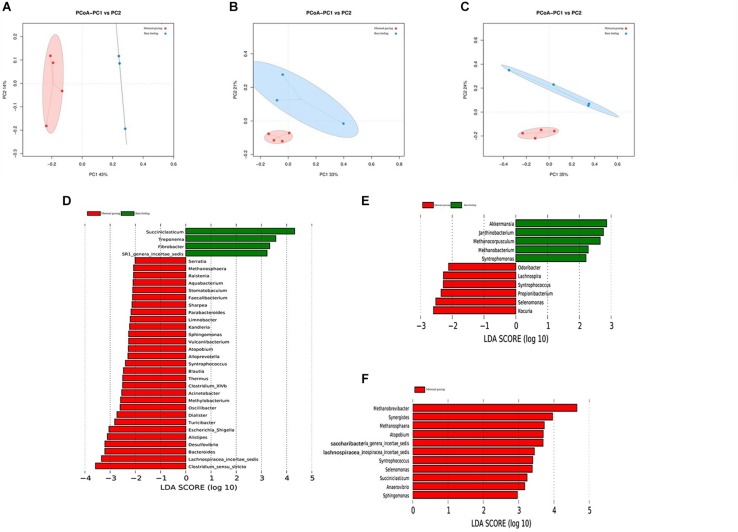
Microbial community difference between the two different feeding strategies (maternal grazing and barn feeding) groups. **(A)** diversity analyses based on the rumen microbiota; **(B)** diversity analyses based on the jejunal microbiota; **(C)** diversity analyses based on the ileal microbiota. **(D–F)** significantly differential genera based on the linear discriminant analysis effect size (LEfSe) cladogram, and the differences are represented by the color of the group.

The gastrointestinal microbiota, including the microbiota in the rumen, jejunum, and ileum, were further analyzed. According to the alpha diversity analyses, chao1 indexes indicated that barn feeding with the starter feed and alfalfa hay were beneficial to the diversity of the gastrointestinal microbiota (Supplementary Table S8). Moreover, the beta diversity analyses revealed that the compositions of the gastrointestinal prokaryotic community of the yak calves in two different feeding groups were significantly different (Figures 3A–C).

By using LEfSe analyses, differential microbiota were further identified based on the counts of different microbes (Supplementary Figure S2 and Figures 3D–F). A total of 33 differential ruminal genera were identified between the two different feeding groups; among these, *Succiniclasticum*, *Treponema*, *Fibrobacter*, and *SR1_genera_incertae_sedis* were significantly increased in the maternal grazing group, and *Serratia*, *Methanosphaera*, *Raistonia*, *Aquabacterium*, *Stomatobaculum*, *Faecailbacterium*, *Sharpea*, *Parabacteroides*, *Limnobacter*, *Kandieria*, *Sphingomonas*, *Vulcanlibacterum*, *Atopobium*, *Alloprevotella*, *Syntrophococcus*, *Blautia*, *Thermus*, *Clostridium_XIVb*, *Acinetobacter*, *Methylobacterium*, *Oscilli- bacter*, *Dialister*, *Turicibacter*, *Escherichia/Shigella*, *Alistipes*, *Desulfovibrio*, *Bacteroides*, *Lachnospiracea_incertae_sedis*, and *Clostridium_sensu_stricto* were significantly increased in the barn feeding group (Figure 3D). Additionally, significantly increased genera were identified in the jejunum of the grazing group, including *Akkermansia*, *Janthinobacterium*, *Methanocorpusculum*, *Methanobacterium*, and *Syntrophomonas* and in the jejunum of the barn feeding group, including *Odoribacter*, *Lachnospira*, *Syntrophococcus*, *Propionibacterium*, *Selenomonas*, and *Kocuria* (Figure 3E). Furthermore, a total of 11 significantly increased genera were identified in the ileum of yaks in the barn feeding group, including *Methanobrevibacter*, *Synergistes*, *Methanosphaera*, *Atopobium*, *Syntrophococcus, Selenomonas*, *Succiniclasticum*, *Anaerovibrio*, *Sphingomonas, Saccharibacteria_genera_incertae_sedis*, and *Lachnospiracea_incertae_sedis* (Figure 3F). Furthermore, the similar results which contained the similar but less differential bacteria were also identified by using the Mann-Whitney *U* test (Supplementary Table S9).

## Discussion

Our results indicate that the yak calves in the barn feeding group provided the milk replacer, starter feed, and alfalfa hay during early life showed improved growth performance and organ development, in accordance with the results of previous studies on lambs during early life (Liu et al., 2017; Saro et al., 2018; Yang et al., 2018). These significantly improved growth performance were resulted from the significantly increased daily DMI of yak calves from barn feeding group. Specifically, the lower DMI observed for animals kept in grazing feeding probably means that yak calves have some restriction on dry matter intake when were kept in nursing, which could further influence the rumen fermentation and intestinal nutrients absorption of yak calves.

Similar to a previous study, rumen fermentation characteristics were significantly affected due to the significant differences in nutrient supplementation, which represented the increased carbohydrate fermentation in the rumen (Raghuvansi et al., 2007; Xue et al., 2017; Yang et al., 2018). In the present study, the significantly increased propionate, butyrate, isobutyrate, and valerate represented the significantly increased utilization of energy in the rumen, which indicated increased energy intake and that more nutrients could be provided to increase growth performance and organ development when propionate was absorbed and converted to glucose, amino acids, and lipids (Orskov, 2012). Specially, a higher molar proportion of propionate may indicate an increased supply of propionate for gluconeogenesis, which is especially important for growing calves. Meanwhile, the significantly increased propionate, butyrate and isobutyrate could be directly used by rumen epithelial cells, promoting ruminal papillae development and further improving the absorption of VFA (Steele et al., 2016). Furthermore, carbohydrate metabolism, energy metabolism, and lipid metabolism of the rumen epithelium were shown to be enhanced in the barn feeding group by using RNA sequencing. These results again proved that the ruminal fermentation and absorption of carbohydrates, lipids, and amino acids were enhanced in the barn feeding group, which could further influence the metabolism and development process.

Furthermore, two different feeding strategies were composed of different nutrition compositions provided to the yak calves, which further induced the differences of intestinal enzymes. In the maternal grazing group, the main nutrient resource of pre-ruminant yak calves was breast milk; thus, the activities of gastrointestinal protease and lipase could be significantly increased to obtain more absorbable nutrients. Hence, the present study found that the duodenal trypsin and lipase, jejunal lipase, and ileal lipase concentrations were significantly increased in the maternal grazing groups. In the barn feeding group, the significant increase in the α-amylase of the duodenum and jejunum was mainly induced by supplementation with the starter feed (Harmon, 2009). The significant increase in trypsin in the jejunum and ileum may have been induced by the increased microbial protein from the rumen (Geiger et al., 2014). Moreover, the significantly increased villus height and crypt depth of the duodenum, jejunum, and ileum in the barn feeding group all indicated that the absorption of the intestine was significantly enhanced, which benefited from the increase in nutrition digestion and was beneficial to the development and growth of yak calves. Overall, significantly enhanced rumen fermentation, intestinal digestion, and gastrointestinal absorption abilities were identified in yak calves in the barn feeding group during early life.

Recently, several studies have focused on differences of gastrointestinal microbiota between wild and domesticated animals, including lizards, cheetahs, yaks, lambs, deer mice, and seals (Nelson et al., 2013; Kohl and Dearing, 2014; Kohl et al., 2017; Wasimuddin et al., 2017; Schmidt et al., 2019). These studies all identified that the diversity and richness of the gastrointestinal microbiota were increased in animals from the wild environment than in captive animals. However, in contrast to these studies, our research compared the diversity and richness of the gastrointestinal microbiota of yak calves from maternal grazing and barn feeding groups during early life. Therefore, our study identified that barn feeding was beneficial to the diversity and richness of gastrointestinal microbiota. As scientifically known, the colonization of the gastrointestinal microbiota in newborn ruminants starts during birth, and newborn ruminants could inherit and share some gastrointestinal microbiota with their mother yaks. In the present study, during early life, the gastrointestinal microbiota was mainly affected by the altered diet and environmental exposure, especially the altered nutritional supplementation. The barn feeding, which supplied the milk replacer, starter feed, and alfalfa hay, could provide enough carbohydrate, protein, and lipid for the growth and proliferation of microbes, which indicated that more microbial species could survive in the gastrointestinal tract due to the abundant sources of carbon and nitrogen (Nelson et al., 2013; Dorrestein et al., 2014). In contrast, a lower dry matter intake of a milk and pasture diet from maternal grazing group cause a lower availability and variety of nutrients in the rumen for microbial growth. Meanwhile, the yaks calves kept in grazing feeding were less stimulated to consume forage in function of presence of their mothers, which also cause a lower rumen abundance and diversity.

A variety of nutrients results in different abundances and functions of microorganisms (Ben Salem et al., 2005; Abecia et al., 2014); moreover, altered gastrointestinal microbiota could further affect the development and metabolism of host animals (Morgavi et al., 2015). As we mentioned before, two different feeding strategies had different abundances of carbon and nitrogen source. Hence, several differential microbes were identified in the rumen and intestine. In the rumen, the significantly increased microbes of yak calves in the maternal grazing group were mostly involved in the utilization of fibrous carbohydrates, including *Treponema*, and *Fibrobacter* (Kudo et al., 1987; Mansfield et al., 1994; Klevenhusen et al., 2017). In contrast, the significantly increased microbes of yak calves in the barn feeding groups were mostly involved in the utilization of non-fibrous carbohydrate, including the *Sharpea*, *Sphingomonas*, *Atopobium*, *Syntrophococcus*, *Clostridium*_*XIVb*, *Acinetobacter*, *Oscillibacter*, *Dialister*, *Desulfovibrio*, *Bacteroides*, *Lachnospiracea*_*incertae*_*sedis*, and *Clostridium*_*sensu*_*stricto*, which have also been proven to be related to increased rumen fermentation in the barn feeding group, especially the increased production of propionate (Lu et al., 2005; Edwards et al., 2017; Granja-Salcedo et al., 2017; Zhang et al., 2017). These results again proved that the different nutrient compositions could lead to different abundances and functions of microorganisms, which could further influence the utilization of nutrients. Specifically, the increase in *Methanosphaera* has indicated that the rumen fermentation of carbohydrates and the hydrogen produced by fermentation were increased, which was in accordance with the rumen fermentation in the present study and proved that more carbohydrates were utilized and more energy was provided in the rumen (Hook et al., 2010; Janssen and Kirs, 2008). Similarly, in the jejunum and the ileum, *Odoribacter*, *Lachnospira*, *Syntrophococcus*, *Propionibacterium*, *Selenomonas*, *Kocuria*, *Synergistes*, *Atopobium*, *Succiniclasticum*, *Anaerovibrio*, *Sphingomonas*, *Saccharibacteria*_*genera*_*incertae*_*sedis*, and *Lachnospiracea*_*incertae*_*sedis* were altered in barn feeding group; these genera were also induced by the more abundant carbohydrates supplied from both fibrous carbohydrate and non-fibrous carbohydrates and have been suggested to be involved in the enhanced intestinal digestion and absorption function observed in the barn feeding groups (McAllister et al., 2011; Ramakrishna, 2013; Li et al., 2016). The increased microbes could produce additional VFAs by using the more abundant carbohydrates supplied from both fibrous carbohydrate and non-fibrous carbohydrates, and the increased VFAs could enhance the immune function of yak calves in the barn feeding groups (Kim Y. H. et al., 2016; Nastasi et al., 2017). Furthermore, the colonization of gastrointestinal microbiota during early life could further influence the subsequent gastrointestinal microbiota of adult ruminants (Ben Salem et al., 2005; Kim Y. H. et al., 2016). Specifically, the effect of differentially supplementing carbohydrates during early life, induced in two different feeding strategies, on the subsequent gastrointestinal microbiota and the related rumen fermentation, intestinal digestion, and gastrointestinal absorption function of adult ruminants have been widely proven (Khan et al., 2016; Meale et al., 2016). Hence, barn feeding yak calves during early life was beneficial to the digestion and absorption function of yaks both during early life and the subsequent adult period, which could further benefit the growth of yaks.

The immune system is stimulated by exposure to microbes, including those from gastrointestinal tract (Imani et al., 2017; Liu et al., 2017; Wang et al., 2017). Hence, the two different feeding strategies could influence the gut microbiota and further influence gastrointestinal immune functions. In the present study, enhanced rumen immune function, which concluded from the increased gene expression in the anti-inflammation and pro-inflammatory regulation related pathways and the immune cell development and differentiation related pathways, could result from increased propionate and butyrate in the rumen fluid (Kim M. et al., 2016; Nastasi et al., 2017). Additionally, in the small intestine, the duodenal IL-1β, the jejunal IL-1β, IL-2, TNF-α, and IFN-γ, and ileal IL-1β were significantly increased in the barn feeding group. Cytokines, as immune regulatory proteins, have an important role in the immune system (Ohtsuka et al., 2006). IL-1β, IL-2, TNF-α, and IFN-γ are secreted by monocytes or macrophages and T helper (Th1) cells and can separately promote proinflammatory or anti-inflammatory activity (Opal and DePalo, 2000; Sun et al., 2000). The increase in these cytokines indicated a significant increase in the intestinal immune function of yak calves in the barn feeding group, which could be induced by the rich supplementation of nutrients to support the development of related intestinal immune functions or by altered intestinal microbiota in the present study (Birchenough et al., 2015; Schirmer et al., 2016). In summary, the barn feeding of yak calves with starter feed and alfalfa hay during early life was beneficial to gastrointestinal immune function, by altering the microbiota.

## Conclusion

Collectively, our study shows that barn feeding of yak calves during the pre-weaning period is beneficial to the gastrointestinal development and its digestion, absorption, and immune function, and the subsequently enhanced growth and immune function of yak calves, which benefited from the increased species and abundances of different microbes that could use different carbon and nitrogen sources from both fibrous carbohydrate and non-fibrous carbohydrate.

## Data Availability Statement

The sequence data were deposited and are available in the Sequence Read Archive (SRA) of NCBI with the accession project numbers PRJNA543073.

## Ethics Statement

All the yak calves and experimental protocols in this study were approved by the Institution Animal Care and Use Committee of the Northwest A&F University (protocol number NWAFAC1118).

## Author Contributions

ZC, SW, SL, and JY conceived and designed the experiments. ZC, SW, SL, LS, and GZ mainly performed the animal feeding experiments and sample collection. ZC, SW, SL, LS, YF, SC, and YC performed the laboratory analysis, SW and ZC analyzed the data. JY, YC, and SL contributed reagents, materials and analysis tools. SW and ZC wrote the manuscript. JY, SW, and SL had primary responsibility for final content. All authors read and approved the final manuscript.

## Conflict of Interest

GZ was employed by Datong Yak Breeding Farm of Qinghai Province.

The remaining authors declare that the research was conducted in the absence of any commercial or financial relationships that could be construed as a potential conflict of interest.

## Footnotes

1 http://drive5.com

2 http://www.ncbi.nlm.nih.gov/tools/primer-blast/

## Abbreviations

DEGs: differentially expressed genes
FPKM: Fragments per kilobase of exon per million mapped reads
IFN- γ: interferon- γ
IL-1 β: inteleukin-1 β
IL-10: inteleukin-10
IL-2: inteleukin-2
IL-4: inteleukin-4
KEGG: Kyoto Encyclopedia of Genes and Genomes
LDA: linear discriminant analysis
LEfSe: linear discriminant analysis effect size
OTUs: operational taxonomic units
PCoA: principal coordinated analysis
qRT-PCR: quantitative real-time PCR
sIgA: secreted immunoglobulin A
TNF- α: tumor necrosis factor- α
VFA: volatile fatty acid.
