# Supplementary material for: From Maternal Grazing to Barn Feeding During Pre-weaning Period: Altered Gastrointestinal Microbiota Contributes to Change the Development and Function of the Rumen and Intestine of Yak Calves
Source: Front Microbiol. 2020 Apr 3;11:485. doi: 10.3389/fmicb.2020.00485 (PMC7145940; doi:10.3389/fmicb.2020.00485)
Supplement: Supplementary file 1 [file Data_Sheet_1.pdf]

Running title: two different feeding strategies alter gastrointestinal microbiota

Title: From Maternal Grazing to Barn Feeding During Pre-weaning Period: Altered Gastrointestinal Microbiota Contributes to Change the Development and Function of the Rumen and Intestine of Yak Calves

Authors: Zhanhong Cui[*, #, ¶], Shengru Wu[*, ¶, 1], Shujie Liu[#, 1], Lu Sun[#], Yuzhe Feng[#], Yangchun Cao[*], Shatuo Chai[#], Guomo Zhang[&], Junhu Yao[*, 1]

Institution: * College of Animal Science and Technology, Northwest A&F University, Yangling, Shaanxi, China

Qinghai Academy of Animal Husbandry and Veterinary Sciences, Qinghai University, Xining, Qinghai, China

[&] Datong Yak Breeding Farm of Qinghai Province, Xining, Qinghai, China

[¶] Co-first author: these authors contributed equally to this work

[1] **Co-corresponding author:**

Shengru Wu, email: wushengru2013@163.com (S. R. Wu); Shujie Liu, email: mkylshj@126.com (S. J. Liu); Junhu Yao, email: yaojunhu2004@sohu.com (J. H. Yao); Tel.: +86 13891883031.

Table S1. Nutrient composition of the alfalfa, starter feed, and milk replacement provided to the yak calves from barn feeding group in the present study.

Table S2. Nutrient content of fresh grass and yak milk for yak calves from maternal grazing group.

Table S3. The concentration and purity of the extracted DNA for further 16S rRNA gene sequencing.

Table S4. Statistical data and quality controlled data of the RNA-Sequencing reads for nine samples.

Table S5. The primer sequences for *GAPDH* (internal control gene) and the tested mRNAs (DEGs selected by RNA sequencing).

Table S6. The differentially expressed mRNAs between two different feeding strategies involved in metabolism related pathways.

Table S7. The differentially expressed mRNAs between two different feeding strategies involved in immune related pathways.

Table S8. The alpha diversity analyses based on the gastrointestinal microbiota of different feeding paradigm (maternal grazing and barn feeding) groups.

Table S9. Microbial community difference between the two different feeding strategies (maternal grazing and barn feeding) groups by using the Mann-Whitney U test.

Figure S1. Effect of two feeding strategies (maternal grazing and barn feeding) during early life on the DMI differences in 9 different measurement.

Figure S2. Significantly differential microbes (phylum, class and order levels) based on the linear discriminant analysis effect size (LEfSe) cladogram, and the differences are represented by the colour of the group.

Supplementary information

Table S1. Nutrient composition of the alfalfa, starter feed, and milk replacement used in the present study.

Items (%)	milk replacer	alfalfa	starter diet
Dry matter (DM)	94.00	93.80	87.90
Crude protein (CP)	24.00	12.50	20.00
Ether extract (EE)	16.00	0.90	4.70
Neutral detergent fiber (NDF)	-	56.45	10.90
Acid detergent fiber (ADF)	-	40.40	4.10
Calcium (Ca)	0.60～3.00	0.98	0.80
Phosphorus (P)	0.50～2.00	0.18	045
Lysine	2.20	0.85	1.06
Methionine	1.00	0.17	0.33

Table S2. Nutrient content of fresh grass and yak milk for yak calves from maternal grazing group.

Pasture		Yak milk	
Wet pasture production (g/m^2)	168.13	Milk production (kg/d)	1.04
Dry matter (DM) (%)	53.24	Total solid constituent (%)	17.45
Crude protein (CP) (%)	10.48	Milk fat (%)	6.58
Ether extract (EE) (%)	2.82	Milk protein (%)	5.03
Neutral detergent fiber (NDF) (%)	49.03	Lactose (%)	4.93
Acid detergent fiber (ADF) (%)	29.53		
Ash (%)	8.48		

Table S3. The concentration and purity of the extracted DNA for further 16S rRNA gene sequencing.

Sample	Concentration (ng/ul)	A260/280
**Rumen Barn feeding 1**	126.4	1.81
**Jejunum Barn feeding 1**	185.3	1.83
**Ileum Barn feeding 1**	106.6	1.85
**Rumen Barn feeding 2**	133.1	1.85
**Jejunum Barn feeding 2**	74.7	1.85
**Ileum Barn feeding 2**	97.3	1.81
**Rumen Barn feeding 3**	126.4	1.83
**Jejunum Barn feeding 3**	78.8	1.82
**Ileum Barn feeding 3**	65.5	1.80
**Rumen Barn feeding 4**	143.2	1.80
**Jejunum Barn feeding 4**	108.7	1.82
**Ileum Barn feeding 4**	69.7	1.85
**Rumen Maternal grazing 1**	89.7	1.85
**Jejunum Maternal grazing 1**	158.3	1.84
**Ileum Maternal grazing 1**	75.8	1.82
**Rumen Maternal grazing 2**	128.2	1.85
**Jejunum Maternal grazing 2**	88.9	1.85
**Ileum Maternal grazing 2**	63	1.83
**Rumen Maternal grazing 3**	91.8	1.85
**Jejunum Maternal grazing 3**	172.5	1.85
**Ileum Maternal grazing 3**	84.7	1.82

Table S4. Statistical data and quality controlled data of the RNA-Sequencing reads for nine samples.

Sample ID	Total Raw Reads(Mb)	Total Clean Reads(Mb)	Total Clean Bases(Gb)	Clean Reads Q20(%)	Clean Reads Q30(%)	Clean Reads Ratio(%)
MG-1	45.42	42.60	6.39	97.42	92.13	93.80
MG-2	52.52	48.94	7.34	97.19	91.58	93.19
MG-3	45.26	42.96	6.44	98.16	94.05	94.92
MG-4	41.04	38.56	5.78	97.47	92.22	93.94
BF-1	47.58	44.07	6.61	97.81	93.18	92.64
BF-2	43.10	40.03	6.00	97.67	92.86	92.88
BF-3	48.60	44.99	6.75	97.72	92.97	92.58
BF-4	47.94	44.68	6.70	98.09	93.94	93.20
BF-5	42.07	39.13	5.87	98.04	93.78	93.02

Table S5. The primer sequences for GAPDH (internal control gene) and the tested mRNAs (DEGs selected by RNA sequencing).

Gene name/abbrevietion	Primer sequences (5'to 3')	Production Size
GAPDH	F: CGACTTCAACAGCGACACTCA<br>R: GGTCCAGGGACCTTACTCCTT	169
SEPW1	F: GGACACGGAGAGCAAGTTTC<br>R: GAGATGAGGGATGGGGAAGG	170
GPX1	F: AACGACGTCTCCTGGAACTT<br>R: GAGGCTGGGATGGATAGGAC	288
ATF3	F: TTAACATCCCGGCCATCCTT<br>R: CTAGAAAGCACAACCCTGGC	246
FOS	F:TTTGACTGCTCGCGATCATG<br>R:CAGATCGGTGCAGTAGTCCT	176
CCL20	F:CATCACAGCACTTCAGAGGC<br>R:ATGTCACAGGCTTCATTGGC	244

Table S6. The differentially expressed mRNAs between two different feeding strategies involved in metabolism related pathways.

Metabolic process	Up-regulated genes (barn feeding verse maternal grazing)	Down regulated genes (barn feeding verse maternal grazing)
Amino acid metabolism	LOC102270060/LOC102281032/ novel_G000288/novel_G000351	GLUL/HMGCS1/novel_G000256
Carbohydrate metabolism	ACSS1/novel_G000130/ novel_G000351/novel_G000793	GLUL/ACSM5/HMGCS1/novel_G00042 /novel_G000699
Energy metabolism	CA4/CA8/NDUFA4L2	GLUL/ATP12A/CA14/novel_G000002/ novel_G000005/novel_G000011
Lipid metabolism	LOC102271037/LOC102271453 LOC102274704/LOC102283454 /novel_G000654	HMGCS1/LOC102284405/ novel_G000085/novel_G000571

Table S7. The differentially expressed mRNAs between two different feeding strategies involved in immune related pathways.

Pathway	Up-regulated genes (barn feeding verse maternal grazing)	Down regulated genes (barn feeding verse maternal grazing)
Intestinal immune network for IgA production	novel_G000651;novel_G000803;novel_G000259;novel_G000263;novel_G000381;novel_G000266;novel_G000264;	novel_G000362;TNFAIP3; novel_G000688; novel_G000380; novel_G000262; novel_G000267; novel_G000392; novel_G000261; novel_G000385
NF-kappa B signaling pathway	novel_G000720; SYNGR3; novel_G000651; novel_G000803; novel_G000259; novel_G000263; novel_G000381; novel_G000266; novel_G000264	TNFAIP3; novel_G000362; novel_G000688; novel_G000380; novel_G000262; novel_G000267; novel_G000392; novel_G000261; novel_G000385
Inflammatory bowel disease	LRRC71; novel_G000259; novel_G000263; novel_G000381; novel_G000266; novel_G000264	novel_G000380; novel_G000262; novel_G000267; IL22; novel_G000392; novel_G000261; novel_G000385
Th17 cell differentiation	RXRG; novel_G000259; novel_G000263; novel_G000381; novel_G000266; novel_G000264	novel_G000380; novel_G000262; novel_G000267; IL22; novel_G000392; novel_G000261; novel_G000385
Th1 and Th2 cell differentiation	novel_G000259; novel_G000263; novel_G000381; novel_G000266; novel_G000264	novel_G000380; novel_G000262; novel_G000267; novel_G000392; novel_G000261; novel_G000385
T cell receptor signaling pathway	novel_G000259; novel_G000263; novel_G000381; novel_G000266; novel_G000264	novel_G000380; novel_G000262; novel_G000267; novel_G000392; novel_G000261; novel_G000385
B cell receptor signaling pathway	novel_G000362; novel_G000254; novel_G000803	novel_G000362; novel_G000688; novel_G000651
NOD-like receptor signaling pathway	LOC102277645; SYNGR3; LOC102279350; LRRC71; GBP5; LOC102283262; LOC102276796	TNFAIP3; LOC102271974; novel_G000421
Natural killer cell mediated cytotoxicity	novel_G000720; novel_G000651; novel_G000803; novel_G000151	novel_G000362; novel_G000688
TGF-beta signaling pathway	PITX2; IRX1	MYC; SMAD9; CRYGN

Table S8 The alpha diversity analyses based on the gastrointestinal microbiota from different feeding paradigms (maternal grazing and barn feeding) groups.

Gastrointestinal tract	Items	Treatments		SEM	*P*-value
		Barn feeding	Maternal grazing		
Rumen	Chao1	1253.68[a]	537.20[b]	100.759	＜0.001
	Shannon_2	7.08	6.34	2.509	0.174
jejunum	Chao1	1190. 20[a]	787. 17[b]	9. 629	0. 027
	Shannon_2	6. 64	5. 80	0. 478	0. 520
ileum	Chao1	468. 60	683. 50	0. 485	0. 517
	Shannon_2	3. 80	4. 02	0. 029	0. 872

Table S9 Microbial community difference between the two different feeding strategies (maternal grazing and barn feeding) groups by using the Mann-Whitney U test.

	taxonomy	Barn feeding	Maternal grazing	P value
Ruminal	*Succiniclasticum*	0.0056±0.0008	0.0500±0.0154	0.007
	Clostridium_sensu_stricto	0.0068±0.0024	0.0000±0.0000	0.050
	Treponema	0.0014±0.0004	0.0089±0.0018	0.002
	Escherichia/Shigella	0.0023±0.0005	0.0000±0.0000	0.024
	Above_genus	0.7979±0.0265	0.5843±0.1093	0.050
	Prevotella	0.0811±0.0222	0.2960±0.1241	0.050
	Fibrobacter	0.0008±0.0002	0.0049±0.0022	0.050
Jejunal	*Treponema*	0.0006±0.0003	0.0052±0.0027	0.060
Ileal	*Turicibacter*	0.0406±0.0113	0.0098±0.0059	0.060
	Methanobrevibacter	0.0793±0.0349	0.0022±0.0011	0.050
	Ruminococcus	0.0083±0.0028	0.0026±0.0011	0.050

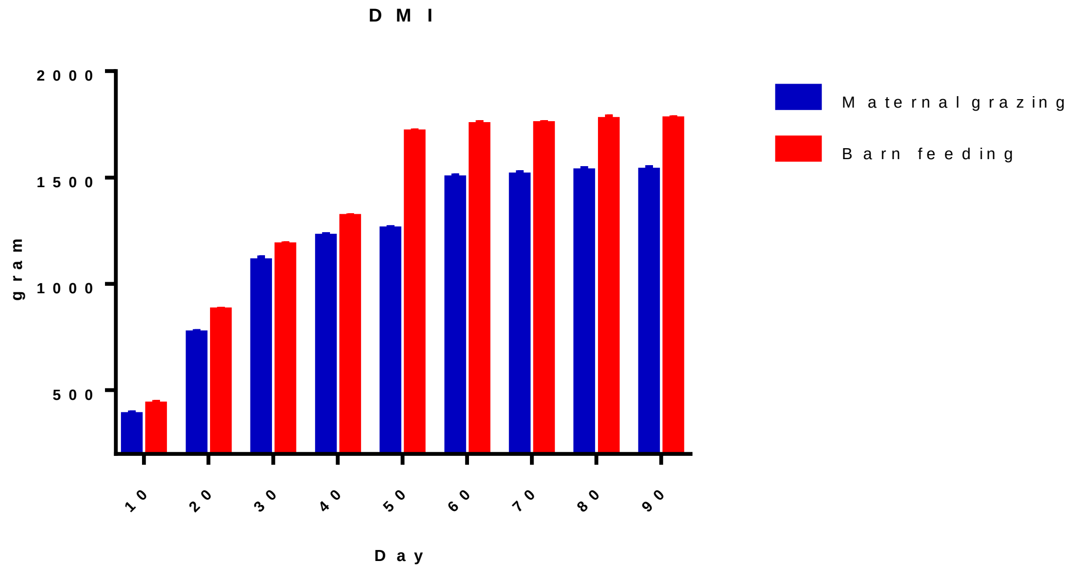

Figure S1. Effect of two feeding strategies (maternal grazing and barn feeding) during early life on the DMI differences in 9 different measurement.

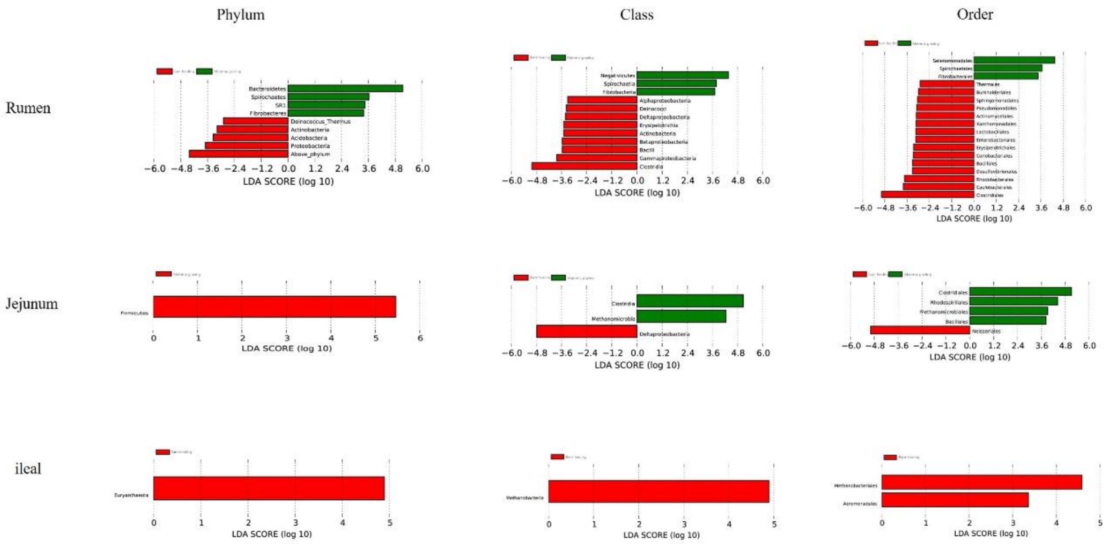

Figure S2. Significantly differential microbes (phylum, class and order levels) based on the linear discriminant analysis effect size (LEfSe) cladogram, and the differences are represented by the colour of the group.
